# Miscellany

**Published:** 1887-12

**Authors:** 


### Miscellany.

---

THE following works will be issued during December by the New York publishers, Leonard & Co., 141 Broadway: "Diseases of Women," a work based upon the practical experience and teachings of the following eminent gynecologists: Drs. Thomas, Munde, Hunter, Lusk, McLane, Skene, Garrigues, Barker, Emmet, etc.; 436 pages; cloth, $1.50. "Diseases of Infancy and Childhood," with over 400 formulæ and prescriptions by Drs. Jacobi, Hammond, Flint, Loomis, Janeway, Bulkley, Agnew, etc.; 300 pages; cloth, $1.00. "Diseases of Heart and Lungs," with over 350 formulæ and prescriptions by Drs. Draper, Delafield, Leaming, J. Lewis Smith, Loomis, Clark, Janeway, etc.; 204 pages; cloth, $1.25.

---

A lady, consulting an eccentric Boston physician, said: "The trouble is, doctor, that I can neither lay nor set." "Then, madam," was the reply, "I would respectfully suggest the propriety of roosting." To which the editor of the "Med. Classic" adds that he evidently had her "fowl."

Dr. Laenger, the chief physician at the Vienna Hospital, attempted suicide recently by morphine. He was promptly treated by the performance of tracheotomy, and the mechanical inflation of the lungs through the opening (Fell's method), and recovered.

---

A Mrs. Partingtonian old lady, being asked her opinion of the relative merits of homeopathy and the regular schools, answered, that for *infantry*, homeopathy might be good enough, but for *adultery*, she preferred the good, old-fashioned doctor.

---

School-teacher to anxious parent: "Your son is bright, intelligent, and getting along well in everything but handwriting." Parent: "That's all right; his writing doesn't matter. I am going to make a doctor of him."

